# Acute admissions to a community hospital - health consequences: a randomized controlled trial in Hallingdal, Norway

**DOI:** 10.1186/s12875-014-0198-1

**Published:** 2014-12-10

**Authors:** Øystein Lappegard, Per Hjortdahl

**Affiliations:** Department of Hallingdal sjukestugu, Medical Clinic of Ringerike General Hospital, Vestre Viken Hospital Trust, Ål, 3570 Norway; Department of General Practice, Institute of Health and Society, University of Oslo, Oslo, Norway

**Keywords:** Patient admission, Emergency health services, General practitioners, Community hospital, Health care systems, Patient outcome assessments

## Abstract

**Background:**

Health care professionals in several countries are searching for alternatives to acute hospitalization. In Hallingdal, Norway, selected acute patients are admitted to a community hospital. The aim of this study was to analyse whether acute admission to a community hospital as an alternative to a general hospital had any positive or negative health consequences for the patients.

**Methods:**

Patients intended for acute admission to the local community hospital were asked to join a randomized controlled trial. One group of the enrolled patients was admitted as planned (group 1, n = 33), while another group was admitted to the general hospital (group 2, n = 27). Health outcomes were measured by the Nottingham Extended Activity of Daily Living Questionnaire and by collection of data concerning specialist and community health care services in a follow-up year.

**Results:**

After one year, no statistical significant differences in the level of daily function was found between group 1 (admissions to the community hospital) and group 2 (admissions to the general hospital). Group 1 had recorded fewer in-patient days at hospitals and nursing homes, as well as lower use of home nursing, than group 2. For outpatient referrals, the trend was the opposite. However, the differences between the two groups were not at a 5% level of statistical significance.

**Conclusions:**

No statistical significant differences at a 5% level were found related to health consequences between the two randomized groups. The study however, indicates a consistent trend of health benefits rather than risk from acute admissions to a community hospital, as compared to the general hospital. Emergency admission and treatment at a lower-level facility than the hospital thus appears to be a feasible solution for a selected group of patients.

**Trial registration:**

ClinicalTrials.gov NCT01069107. Registered 2 April 2010.

## Background

Because of the increasing number of hospital admissions and rising treatment costs, many countries are searching for alternatives to acute hospital admissions [1-3]. In Norway, the Coordination Reform challenges health care providers to develop alternative treatments before and instead of hospitals [4]. The health care system in Norway is divided into two levels. The state is responsible for the specialist health care services, including the hospitals, outpatient services and emergency services. The municipalities are responsible for primary health care, including general practice, home-based care and nursing homes. In a few places, such as in rural areas and in the northern parts of Norway, institutions offer intermediate beds with services between these two administrative levels [5].

Hallingdal Sjukestugu (HSS) can be categorized as a cottage or community hospital that includes an intermediate care unit of 14 beds for patients with somatic diseases. It is run by general practitioners (GPs) under the guidance of specialists at the general hospital. HSS is located in a rural region in Southern Norway with a population of 20,000. HSS is organizationally linked to the nearest hospital, Ringerike General Hospital (RS), 170 km away. There are approximately 600 admissions annually to HSS, with a mean length of stay of 4.8 days (2012). The patients can be divided into three groups: acute admissions, follow-up treatment after general hospital admissions and rehabilitation; each group about equal in size.

The health services at HSS were described in a previous article [6]. When admitting patients to acute care the GPs of Hallingdal must consider RS, HSS or local nursing homes in accordance with the patients’ need for competence and level of care [7]. The region has not developed any “hospital at home” services.

GPs who intend to refer a patient acutely to HSS must first obtain consent from the specialist on call at the general hospital. The list of diagnoses and clinical conditions relevant for acute admissions to HSS has been developed and agreed upon by the combined medical staff at RS and HSS. Patients eligible for acute admissions to HSS are stable with clarified diagnosis or patients who need observation or basic investigation and who are not critically ill (Table 1).

**Table 1:** **A section from the guidelines for acute admissions to the community hospital (HSS)**

**Main rule**	Patients in need of observation and treatment with frequent supervision by nurses and physicians, but who are not in need of the hospital's specific expertise and equipment
**Samples and clarifications:**	
Observations	Light concussion (unconsciousness < 5 min., no focal neurological findings, GCS 14-15 and without special risk factors); this according to the Scandinavian guidelines where CT is not available
	Fractures and injuries where it is appropriate to take X-rays on the HSS or where further admission to hospital has to be clarified
	Intoxication (alcohol and tablets) after treatment at a municipal emergency unit. Deliberate self-harm should be admitted to RS
	Observation of other causes where hospitalization is not necessary
Treatment and medical follow-up	Patients with infections who do not meet the SIRS criteria for sepsis. If so, the patient will be assessed for hospital admission in consultation with the specialist on call
	COPD exacerbations where treatment has been clarified
	Dehydrated patients who require intravenous fluid therapy
	Hyperemesis
	Nutritional deficiencies
	Blood transfusions
Adjustment of ongoing medical treatment	Diabetes, with both tablet and insulin regulation. Patients with ketoacidosis, hyperglycaemia and with the risk of diabetic coma must be sent to RS
	Heart failure
Palliative and terminal care	Especially concerning complex conditions and younger patients
Emergency deliveries	In cases where the general hospital cannot be reached

There is limited international research about acute admissions outside general hospitals. Critical questions are often raised concerning perceived quality, health outcomes and health economics of such admissions. The present study aims at evaluating whether acute admissions to HSS as an alternative to the general hospital have health consequences for the patients.

## Methods

The study was designed as a randomized controlled trial (RCT). Originally, the project was planned with readmissions as the primary outcome, and the sample size calculations required 70 patients in each group. However, early in the project period it became obvious that this number of patients was unobtainable. Readmissions as the primary outcome was abandoned. The study’s power was calculated with alternative primary outcomes, including health economics, patient satisfaction and patient functional level. All of these, however, indicated a sample size larger than what could be expected from the inclusion rate. The project was still continued with the secondary outcomes as planned and with a triangulation of health outcomes, patients’ perceived quality and health economics as the three main parameters. Due to practical reasons the inclusion period was limited to two years. The present paper analyses the health outcomes, while a previous article focused on the patients’ perceived quality [8]. A third article will discuss the topic of health economics related to the project.

The inclusion criteria were that the patient’s clinical status had to be in accordance with the guidelines for an acute admission to HSS (Table 1), that the GP had consent from the specialist on call at RS and that the patient was a resident of one of the six municipalities in Hallingdal. The exclusion criteria were patients with injuries and acute illnesses in need of diagnosis, treatment or monitoring at a general hospital, births or psychiatric disorders. Mortality and the number of acute transfers from HSS to RS were continually monitored to allow the project to be stopped if critical incidents occurred.

Eligible patients were asked if they were willing to participate in a project related to place of admission. After consent was obtained, a computerized random number generator performed the randomization procedure, where about half of the patients were admitted to HSS (group 1) and the others to RS (group 2). A total of 27 GPs from six municipalities in the Hallingdal region included patients. The research project was approved by the Regional Committee for Medical and Health Research Ethics in Norway (REK) (ref. 2009/1300). The RCT was in compliance with the principles of the Declaration of Helsinki and the Belmont report.

The two groups were compared with regard to their socio-demographic profile and health status. REK gave permission to register the age and length of stay of all patients acutely admitted to HSS during the two-year registration period, to function as a reference group for the selected group of patients entered into the study.

Health outcomes were assessed in two ways. Firstly, the patients’ function in the form of “activities of daily living” (ADL) during the index admission and one year after admission was evaluated. Four trained research assistants at HSS and RS, who interviewed the patients and/or their family members, obtained the information. The measuring tool was a validated Norwegian translation of the Nottingham extended ADL scale (NEADL), an internationally widely used questionnaire [9-11].

Secondly, the patients’ health status was assessed by collecting data regarding mortality, transfer to the general hospital within two days of the index admission, readmissions and the consumption of both specialist and primary health care services during the first year after the index admission.

The first author obtained information from the specialist health care services extracting relevant information from the shared electronic medical records of RS and HSS. This included the length of in-patient visits to hospitals and the number of out-patient consultations and X-ray examinations.

Health information from the community services was obtained through inquiries to each patient’s GP and to the local health care services. Data were collected through summaries from local record systems or copies of the patients’ records and included the number of in-patient days at the municipal nursing homes and the number of hours for which the patient had received home care services in the form of home nursing or practical assistance. Medical consultations were also registered during the one-year period, including GP consultations, home visits and emergency room consultations, as well as those consultations where the patient only had contact with a nurse at the doctor’s office.

Statistical calculations were performed on the difference between the two groups using IBM SPSS Statistics Version 19. The significance level was set at 5% for all tests.

## Results

**Figure 1:**
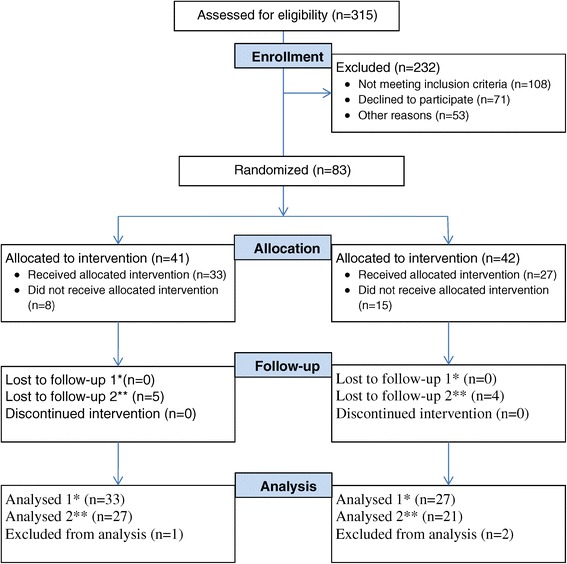
**Flow diagram for the randomized controlled trial.** *Follow-up 1 and Analysed 1 refer to the study of the consumption of health care services. **Follow-up 2 and Analysed 2 refer to the Nottingham EADL study.

Of the 315 patients whom GPs intended to admit to HSS during the two-year period from 1 May 2010, 60 patients (19%) were included in the study (Figure 1). One hundred and eight patients (34%) did not meet the inclusion criteria, mainly because they were not residents of Hallingdal or that they were not competent to provide informed consent. In addition, 53 patients (17%) were not included either because they were not asked by the admitting GP or because of other reasons. A further 71 patients (23%) declined to participate in the project. Because of research ethics requirements we were not allowed to ask these patients their reasons for declining. However, we were allowed to ask the admitting GPs for their general impression of why the patient did not want to participate. Almost without exception, the patients did not want to take the chance of a random allocation to the treatment facility, having a strong desire to be admitted to HSS and not RS. Three main reasons were given for this: they wanted to avoid an arduous and lengthy ambulance trip; the hospital was associated with feelings of stress and hectic activity; and the patients recalled positive experiences with previous admissions to HSS.

After the randomization, 15 patients (5%) withdrew from the project. For ten of these the GP reported the same unwillingness among the patients to be admitted to RS. Eight patients were excluded because of practical reasons, such as lack of available beds or lack of an ambulance.

The 60 patients included in the study were randomized into two groups, with 33 patients in group 1, admitted to HSS, and 27 in group 2, hospitalized at RS. There were no statistical significant differences between the two groups regarding sex, age, length of stay, mortality or readmissions (Table 2). Two patients in group 1 were transferred to RS within two days because of disease progression. The number of chronic diseases averaged 1.7 in group 1 and 1.5 in group 2. The number of different prescription medications used prior to admission averaged 6.1 in group 1 and 5.1 in group 2. Differences between the two groups could not be identified in ethnicity, in the distribution of pensioners and employees, first language, highest education completed or how often they had been admitted to HSS or hospitalized during the two years prior to the index admission. The study had no information about socioeconomic status, or if the patient lived alone or had someone to take care of them.

**Table 2:** **Comparison between patients admitted to the community hospital (HSS) and to the general hospital (RS)**

	**Group 1 (admitted to HSS)**	**Group 2 (admitted to RS)**	**P value**
	**(** ***n*** **= 33)**	**(** ***n*** **= 27)**	
Mean age (years)	71.5 SD = 18.8	71.2 SD = 18.7	0.945^a^
Mean length of stay (days)	5.1 SD = 3.9	5.3 SD = 3.9	0.813^a^
Male	14 (42.4%)	15 (55.6%)	0.311^b^
Deaths within 30 days of admission	3 (9.1%)	2 (7.4%)	1.000^c^
Readmissions <30 days	4 (12.1%)	2 (7.4%)	0.681^c^

SD, standard deviation.

^a^Two-sample t test (equal variance assumed).

^b^Chi-squared test.

^c^Fisher exact test.

The admission diagnoses in the two groups were pneumonia (18), infection of the urinary tract (6), other infections (6), trauma without need for surgical treatment (6), chronic diseases with exacerbations (5), various pain conditions (4), dehydration (3), cancer patients with a worsening of symptoms (2) and a broad specter of other diagnoses (10).

The age and length of stay of the 60 enrolled patients were compared with all acute admissions to HSS during the two-year period, with the exception of those who met the exclusion criteria. The mean age of the enrolled patients was 71.1 years (SD = 19.1), and of the reference group, 73.6 years (SD = 19.9) (two-sample t test, p = 0.421). The mean length of stay for the enrolled patients was 5.1 days (SD = 3.8), and for the reference group, 4.6 days (SD = 4.1) (p = 0.487).

At the end of the follow-up period, there were complete results from the NEADL questionnaire for 27 out of 33 patients in group 1 and for 21 out of 27 patients in group 2. Missing data were attributable to deaths (nine patients) or practical difficulties in obtaining the information (three patients). The patients’ functional level as reported by the NEADL assessment was given by a mean score prior to the index admission of 43.96 for group 1 and 40.43 for group 2, on a scale where the maximum score at full functional level is 66. Assuming equal variances in the two groups, no significant difference in the level of daily function was found (t test, SD = 4.450, p = 0.431). One year after discharge, group 1 had increased the mean score by 1.93 to 45.89, while group 2 had decreased the mean score by 1.86 to 38.57. Assuming equal variances in the two groups, a t test showed no significant difference in the change in function between the two groups (SD = 2.223, p = 0.095).

Patients’ use of health services in the follow-up year after discharge from HSS or RS is summarized in Table 3. The differences between the two groups were not statistical significant either in the number of in-patient days, or in the referrals to out-patient units and radiology examinations as a whole during the follow-up year. There was a significant difference in the number of referrals to the out-patient clinic at HSS: patients in the group initially admitted to HSS were often referred to the out-patient clinic at the same facility.

**Table 3:** **Consumption of specialist and municipal health care services for one year after discharge**

	**Group 1 (** ***n*** **= 33) admitted to HSS**			**Group 2 (** ***n*** **= 27) admitted to RS**			**P value** ^**a)**^
	**Total number**	**Mean per patient**	**SD**	**Total number**	**Mean per patient**	**SD**	
*Specialist health care services*							
In-patient days (total number)	245	7.4	12.1	300	11.1	22.1	0.872
Number in-patient days at HSS	118	3.6	7.0	156	5.8	11.7	0.621
Number in-patient days at RS	117	3.5	6.4	131	4.9	11.4	0.813
Number in-patient days other hospitals	10	0.3	1.0	13	0.5	1.5	0.952
Out-patient/X-ray consult. (total number)	70	2.1	3.0	37	1.4	2.3	0.201
Out-patient/X-ray consultations at HSS	26	0.8	1.4	6	0.2	0.5	0.044*
Out-patient/X-ray consultations at RS	29	0.9	1.3	16	0.6	1.0	0.318
Out-patient/X-ray consult. other hospitals	15	0.5	0.9	15	0.6	1.4	0.752
*Municipal health care services*							
Nursing homes (days)	355	10.8	30.0	524	19.4	50.0	0.078
Home care total (hours)	2415	73.2	136.2	3019	111.8	245.6	0.796
Home nursing (hours)	1983	60.1	117.6	2669	98.9	227.4	0.856
Practical help (hours)	432	13.1	26.4	350	13.0	24.0	0.970
GP office consultations (number)	470	14.2	11.8	352	13.0	13.3	0.562
GP consultations (number)	263	8.0	7.2	193	7.2	7.6	0.612
Nurse consultations (number)	207	6.3	7.4	159	5.9	7.4	0.976

SD, standard deviation.

^a^Independent-samples Mann–Whitney U Test.

*Significant at a 5% level.

Regarding municipal health services, there was a trend of lower usage of nursing homes and home nursing by patients in group 1, but none of the differences were statistical significant. The mean length of stay in the nursing homes for patients in group 1 was 33 days and it was nine days for group 2. There was no significant difference between the two groups in their use of general practice services in the follow-up year (p = 0.562).

## Discussion

Proper treatment at the right place and at the right time is one of the main goals of the Coordination Reform in Norway [4]. This will also be the main issue internationally concerning admissions to intermediate units before, instead of and after hospital treatment. Are the right patients receiving this kind of health service? This can be evaluated by monitoring the patients during their stay, or by analysing health outcomes for groups of patients after their stay. Our research project did not focus on individual monitoring of the patients during their index admission. The modest numbers of transfers from HSS to the general hospital within two days of admission, however, did indicate that the GPs selected the appropriate patients for admission to the intermediate unit. In Great Britain there have been efforts to develop clinical criteria for admissions to cottage hospitals [12]. At community hospitals there are under development systems for monitoring patients during their stay [13]. ALERT is a multi-professional course to train staff in observation skills, developed at Portsmouth Hospital in England and now widely used in Norway [14].

In our study, we retrospectively assessed health outcomes for a group of patients admitted for acute reasons to a community hospital. On ethical grounds, the use of an RCT as a research method may be questioned, as half of the patients were subject to an additional two-hour ambulance ride to the hospital. These ethical aspects were discussed in the project proposal to which the REK gave its approval. Other projects have rejected the use of an RCT because of long transport distances being involved [15]. The main argument for our use of the RCT study design was that the group admitted to the general hospital received medical service matching the normal procedures for emergencies in rural Norway. Ten percent of the Norwegian population lives more than one hour of transportation time from their local general hospital, and very few have a local health service like HSS [16].

The research project had a low acceptance rate, because a relatively large number of patients wanted to be sure of admission to HSS and so declined to participate. Retrospectively, this can be used as an argument in the ethical considerations questioning RCT as the correct research method in a health care study like ours. On the other hand, the fact that more than one quarter of the patients expressed such a strong preference for admissions to HSS can be viewed as an important indication of trust in the local health services. Indirectly, it is also a signal that the patients feel confident that the medical care provided at the intermediate unit is adequate.

In this study we have not statistically tested the similarity between the groups, but the differences. We cannot conclude similarity between groups if there is no evidence of statistically significant differences. We use the tendencies of differences to explore underlying factors that may cause such differences.

The low number of participants implies a limitation in the conclusions that can be drawn from the study. There is a risk of type II error; we do not detect the differences that actually exist. The issue is an illustration of the challenges facing smaller, decentralized units to produce solid research results. This is probably one reason why there is little international research in this field. Both multi-center research projects and an approach through meta-analyzes can provide opportunities for more robust results in the future. What makes the Hallingdal study of general interest is the clear trend of the main results pointing in the same direction, indicating that such a community hospital can function equally well as the general hospital in regard to this group of selected patients.

The NEADL is a well-tested evaluation tool covering an adequate range of everyday functions and was chosen because the data collection phase was considered feasible when interviewing acutely ill patients [17]. Due to the same reason, more comprehensive quality of life tools were not chosen. The NEADL score reflects the degree to which the patient is able to live independently, an important factor when assessing the need for care [18]. One year after discharge from the index admission, there was a difference of 7.32 points in the NEADL score between the two groups in our study. A British survey, using the NEADL to compare the follow-up of elderly patients in community hospitals with that in general hospitals, found a difference of 3.27 points in favour of the group treated in the community hospitals [19]. This was considered to be in the lower range of a clinically relevant difference. In our study, the number of included patients was small, and major changes by a few patients could have a significant impact. The difference in the level of daily function after one year, however, leaves the impression that the group admitted to HSS came out somewhat better than the group admitted to RS, confirming the results of the British study.

Another British study compared health outcomes for elderly patients after acute admission to a community hospital with those for acute admission to a general hospital [15]. The groups were examined six months after admission with a focus on quality of life, readmissions and mortality. The study found no significant differences between the groups, and the authors concluded that community hospitals could be used as alternatives to general hospitals for a variety of types of emergency admissions for elderly patients.

A Norwegian study by Garåsen et al. found that patients who received follow-up care at a lower-level facility after initial hospital treatment had fewer readmissions than patients who had the complete treatment and follow-up at the hospital [20]. In our study, focusing on acute admissions, we found no statistically significant differences between the two groups concerning readmissions or hospital in-patient days during the follow-up year. Nevertheless, it is interesting to note that number of in-patient days in group 1 was consistently lower than in group 2 during the following 12 months, indicating that the HSS group seemed to have health outcomes at least as good as the hospital group.

For the out-patient visits and X-ray consultations, the situation was reversed: the results showed an almost consistent trend of there being fewer in group 2 than in group 1. This may be an indication that patients admitted to hospital underwent more examinations during their stay, whereas these examinations were undergone in the period after discharge for patients admitted to HSS. Only the out-patient clinic at HSS showed a statistically significant difference in the use of such services between the two groups. It may seem more natural for the doctors at HSS, the GPs and the patients themselves to ask for out-patient follow-up locally when the admissions occur locally.

Garåsen et al. also found that patients who received follow-up treatment at a lower-level facility than a hospital were more independent of municipal care [20]. Our findings indicate a similar pattern, with group 1 having health outcomes at least as good as group 2, but the differences between the two groups were not statistical significant. One possible interpretation is that the general hospital chose a nursing home as a standard solution at discharge, whereas HSS collaborated to a greater extent with the municipality to find other local forms of follow-up treatment. Such an interpretation is supported by a shorter mean length of stay in the nursing home for group 2, possibly because some patients were quickly transferred to other health care services in the municipality.

## Conclusions

No statistically significant differences at 5% significance level were found in health outcomes or changes in activities of daily living during a 12-month follow-up period between groups admitted for acute reasons to a community hospital and to a general hospital. The actual findings, however, were consistent in their direction, indicating a trend of health benefits rather than risks for acute admissions to the community hospital compared to the general hospital. This gives an indication that emergency admission and treatment at a lower-level facility than a general hospital can be a viable solution for a selected group of patients. Further research should be carried out to confirm this finding and methods should be developed to monitor patient trajectories to ensure that they are admitted to and treated at the correct level of care.

## Abbreviations

ADL: Activities of daily living
GP: General practitioner
HSS: Hallingdal Sjukestugu, a community hospital in Ål, Hallingdal, Norway
IBM: International business machines, an American multinational technology corporation
NEADL: Nottingham extended activity of daily living scale
RCT: Randomized controlled trial
REK: Regional committee for medical and health research ethics in Norway
RS: Ringerike Sykehus, a general hospital in Hønefoss, Norway, 170 km. from Hallingdal sjukestugu
SPSS: Statistical package for the social sciences, a software package used for statistical analysis
